# The Cascade of Care for Infectious Diseases in Newly Arrived Refugees

**DOI:** 10.3390/ijerph23020229

**Published:** 2026-02-11

**Authors:** Mie Fryd Nielsen, Jane Agergaard, Rebecca Vigh Margolinsky, Line Kibsgaard, Mette Holm, Anne Mette Hvass, Christian Wejse

**Affiliations:** 1 Department of Infectious Diseases, Aarhus University Hospital, Palle Juul-Jensens Boulevard 99, Indgang D3 og E, 8200 Aarhus, Denmark; au18186@clin.au.dk (J.A.); remarg@clin.au.dk (R.V.M.); 2 Department of Forensic Psychiatry, Aarhus University Hospital, Palle Juul-Jensens Boulevard 99, 8200 Aarhus, Denmark; 3 Department of Dermatovenereology, Aarhus University Hospital, Palle Juul-Jensens Boulevard 67, Indgang F, Plan 2, 8200 Aarhus, Denmark; linekibs@rm.dk; 4 Pediatrics and Adolescent Medicine, Aarhus University Hospital, Palle Juul-Jensens Boulevard 99, 8200 Aarhus, Denmark; mette.holm@skejby.rm.dk; 5 Department of Social Medicine & Rehabilitation, Central Denmark Region, Regional Hospital Gødstrup, Hospitalsparken 15, 7400 Herning, Denmark; amhvass@au.dk; 6 Clinic for Complex, Cross-Cultural Medicine, Department of Infectious Diseases, Aarhus University Hospital, Palle Juul-Jensens Boulevard 99, 8200 Aarhus, Denmark; 7 Center for Global Health (GloHAU), Aarhus University, Vennelyst Blvd. 4, 8000 Aarhus, Denmark

**Keywords:** migrant health, refugees, screening, infectious disease

## Abstract

**Highlights:**

**Public health relevance—How does this work relate to a public health issue?**

- Newly arrived refugees are routinely screened for infectious diseases, yet little is known about what happens after a positive screening result and whether individuals successfully complete the cascade of care.
- Loss to follow-up after screening represents a critical gap in infectious disease control and refugee health, with potential consequences for both individual health outcomes and population-level disease prevention.

**Public health significance—Why is this work of significance to public health?**

- The study demonstrates that even in a high-income country with systematic post-arrival screening, substantial attrition occurs after diagnosis, limiting the effectiveness of screening programmes.
- Findings highlight structural and organisational barriers within healthcare systems that may undermine equity in access to diagnosis, treatment, and long-term follow-up for refugee populations.

**Public health implications—What are the key implications or messages for practitioners, policy makers and/or researchers in public health?**

- Screening programmes must be accompanied by strengthened systems ensuring continuity of care, including clear referral pathways, consistent guideline adherence, and systematic use and documentation of professional interpreters.
- Policymakers and researchers should prioritise interventions that address social, organisational, and communication-related barriers to follow-up in refugee health, in order to maximise the public health benefit of post-arrival screening initiatives.

**Abstract:**

(1) Background: Post-arrival screening for infectious diseases is routinely offered to newly arrived refugees in Denmark, including tests for hepatitis B virus (HBV), hepatitis C virus (HCV), human immunodeficiency virus (HIV), and syphilis. This study aimed to examine the cascade of care following positive screening results in a local cohort of refugees in Denmark, with a focus on subsequent clinical management, follow-up, and outcomes. (2) Methods: This retrospective cohort study included 1506 newly arrived refugees of all ages and countries of origin. All were offered a post-arrival infectious disease screening in Denmark. Clinical records were reviewed to assess progression through the cascade of care, including referral, evaluation, follow-up, and clinical outcomes among individuals with positive screening results. (3) Results: Of the 1506 screened refugees, 33 (2.2%) had at least one positive screening result. Among the 15 individuals with detectable hepatitis B surface antigen, six (43%) attended regular follow-up, while eight (57%) were lost during the cascade of care. Two participants screened positive for HCV antibodies; both underwent initial clinical evaluation, but their subsequent care trajectories differed due to repeated non-attendance or undocumented reasons. Only one participant with non-specific syphilis antibodies completed follow-up in accordance with national guidelines. One participant was diagnosed with HIV and successfully linked to care. (4) Conclusions: The prevalence of screened infectious diseases in this local Danish refugee cohort was low and consistent with findings from comparable settings. Although post-arrival screening facilitates the identification of infectious diseases, substantial loss to follow-up occurred after initial diagnosis, limiting the effectiveness of follow-up and treatment. These findings highlight the need for targeted, interdisciplinary strategies addressing organisational, social, and individual barriers to improve continuity of care following screening.

## 1. Introduction

Global forced displacement has increased substantially over the past decades [1], and the health and care needed by this marginalised group is often neglected [2]. Refugees constitute a particularly vulnerable subgroup due to pre-migration exposure, precarious migration trajectories, and resettlement-related challenges [3]. Unlike migrants, whose movement is primarily voluntary, refugees are displaced due to conflict, persecution, or humanitarian crises, and therefore face distinct legal, social, and health-related vulnerabilities [4]. These factors contribute to an increased burden of both communicable and non-communicable diseases [5] compared with host populations, as well as heightened risks of unmet healthcare needs following arrival [6].

Consequently, several countries, including Denmark [7], offer post-arrival health assessments targeting selected infectious diseases such as hepatitis B virus (HBV), hepatitis C virus (HCV), human immunodeficiency virus (HIV), and syphilis [8,9]. While such screening initiatives are effective in identifying previously undiagnosed conditions [7,10] and are generally well accepted among refugees [11], their impact depends not only on case detection but also on successful progression through subsequent steps of care [4,12].

This progression is commonly conceptualised as the cascade of care, a model used for describing and evaluating the care provided by a healthcare system [8]. Breakdowns at any stage of this cascade may compromise individual health outcomes and undermine the public health benefit of screening programmes [11]. Previous studies have reported substantial loss to follow-up after initial screening, yet empirical data describing where and why refugees disengage from care remain limited, particularly in a Danish context.

In Denmark, responsibility for post-arrival health assessments lies with individual municipalities, resulting in marked local variation in scope, organisation, and systematic execution [13]. Unlike many municipalities, Aarhus has offered structured and systematic health assessments to newly arrived refugees since December 2013 [7]. This local initiative includes screening for HBV, HCV, HIV, and syphilis, providing a unique opportunity to examine not only disease prevalence but also the continuity of care within a defined municipal healthcare setting.

The present study examines a local cohort of newly arrived refugees screened for HBV, HCV, HIV, and syphilis in Aarhus, Denmark. The objectives were to (i) estimate the prevalence of these infectious diseases within the cohort and (ii) assess progression through the cascade of care following a positive screening result. Specifically, the study evaluates referral, attendance at clinical evaluation, follow-up according to national guidelines, and the documented reasons for attrition. By identifying critical points of loss to follow-up, the study aims to inform targeted improvements in the organisation and delivery of post-arrival infectious disease care for refugees.

## 2. Materials and Methods

### 2.1. Data from the AARHAUS Study

From January 2014 to January 2020 health assessments, consisting of medical history, a physical examination, and blood samples, were offered to 1506 newly arrived refugees in Aarhus, Denmark. The assessments were performed as a part of the AARHAUS study (Aarhus Refugee Health Assessments Using Systematic approaches) [7]. Participants arrived through three pathways: “quota refugees”, defined as refugees arriving through the quota system of The UN Refugee Agency; “asylum seeking refugees”, defined as refugees with asylum seeking backgrounds; and “family reunited refugees” defined as immigrants becoming reunited with family. The three groups are hereafter collectively referred to as “type of migration,” and the overall study population is referred to as “refugees.” All age groups were included. The practical aspects of the health assessments and inclusion criteria along with information about consent and permissions are described elsewhere. (Hvass et al.) [7]

A variety of screening tests were performed: (i) HBs-Ag detection, (ii) antibodies against HCV, (iii) HIV antibodies against HIV-1, HIV-2 and p24 antigen, or (iv) detection of syphilis non-specific antibodies through Wassermann reaction (WR) or rapid plasma regain (RPR). The tests were performed on blood samples. If venous punctures proved difficult, the blood samples were only repeated after consultation with a medical doctor. The results for tuberculosis screening are described elsewhere [14].

The cascade of care for patients who had positive screenings in any of the tests was evaluated in terms of referral, attendance at clinical evaluation and follow-up.

In addition to infectious disease screening results, baseline data on sex, age, country of origin, educational attainment, and migration status were extracted from the AARHAUS study. Age was recorded at the time of the health assessment. Participants aged 18 years or older were classified as adults, whereas those younger than 18 years were classified as children. The country of origin was defined according to participants’ self-reported country of origin. All participants who had received a residence permit within the last three years and who agreed to participate in the aforenamed health screening were included [7].

### 2.2. Statistical Analysis

The storage of the data is described elsewhere [7].

Because only 33 screened individuals had a positive result for an infectious disease, no statistical analyses were performed on this subgroup. Given the very small number of positive cases, we, as authors, chose not to present disaggregated data in order to avoid potential breaches of data protection and confidentiality regulations. The cascade of care was described descriptively based on information extracted from electronic medical records.

### 2.3. Data from Electronic Medical Records

In Denmark every patient has a unique personal social security number referred to as a CPR number, which is used as an ID number. When in contact with the hospital system in Denmark, the documentation provided by healthcare personnel is linked to the patient’s CPR number in specific electronic medical systems depending on which region the patient is seen. In the Central Region of Denmark, information is stored in the “Electronic Patient Journal” (EPJ) and data are available by healthcare providers involved in the hospitalisation.

The following data was collected from EPJ: (i) whether a referral to evaluation was received; (ii) whether the patient showed up to an evaluation at the hospital clinic; (iii) whether the patient was diagnosed by paraclinical test; and (iv) if any treatment was commenced/initiated and if so, what kind. We compared national guidelines to each medical record of the enrolled participants, to investigate the adherence to these guidelines. The correlation between the two indicated a sufficient cascade of care. The guidelines regarding supplementary tests and examinations on each infectious disease can be found in Table 1. We also used the electronic medical records to find data on compliance/adherence to treatment (a measurable virus was used as an indicator for non-adherence to treatment) and outpatient controls, follow up and outcome along the cascade of care. Data was collected retrospectively by February 2022.

As the infectious diseases of interest in this study require different procedures, the information extracted from the electronic medical files relied on what screening was positive.

For individuals with detected HBs-Ag or HIV antibodies against HIV-1, HIV-2 or p24 antigen, we investigated if the patient had ongoing appointments in the outpatient clinic, as infections with HBV and HIV are chronic and therefore require lifelong control. Furthermore, we investigated whether treatment was prescribed.

For participants with antibodies against HCV, we investigated if treatment was prescribed in case HCV-RNA was detected. As HCV is a disease often found in drug users, we searched for any data on reinfections.

With regard to participants with a positive screening test of syphilis, we investigated whether supplementary tests (Table 1), and antibiotics were offered according to the clinical stage [15]. As recommended by the International Union Against Sexually Transmitted Infection (IUSTI), participants that were syphilis positive were advised to consult the venereology outpatient clinic three, six and twelve months after the treatment had been provided [15].

During the execution of this study, the authors used ChatGPT (software GPT-5.2) for language revision. After using this tool, the authors reviewed and edited the content as needed and therefore take full responsibility for the content of the publication.

## 3. Results

A total of 1506 refugees underwent the health assessment. Participants originated primarily from Western Asia (67.1%), predominantly Syria (64.2%). Women constituted 46.2% of the cohort. Among all participants, the mean age was 22 years, and age ranged from 0 to 76. (Table 2).

Overall, 33 individuals (2.2%) had a positive screening result for at least one infectious disease: hepatitis B virus (HBV; *n* = 14), hepatitis C virus (HCV; *n* = 10), HIV (*n* = 1), or syphilis (*n* = 10). Three individuals screened positive for more than one infection. One initial positive syphilis screening was later refuted by confirmatory testing. 

**Figure 1:**
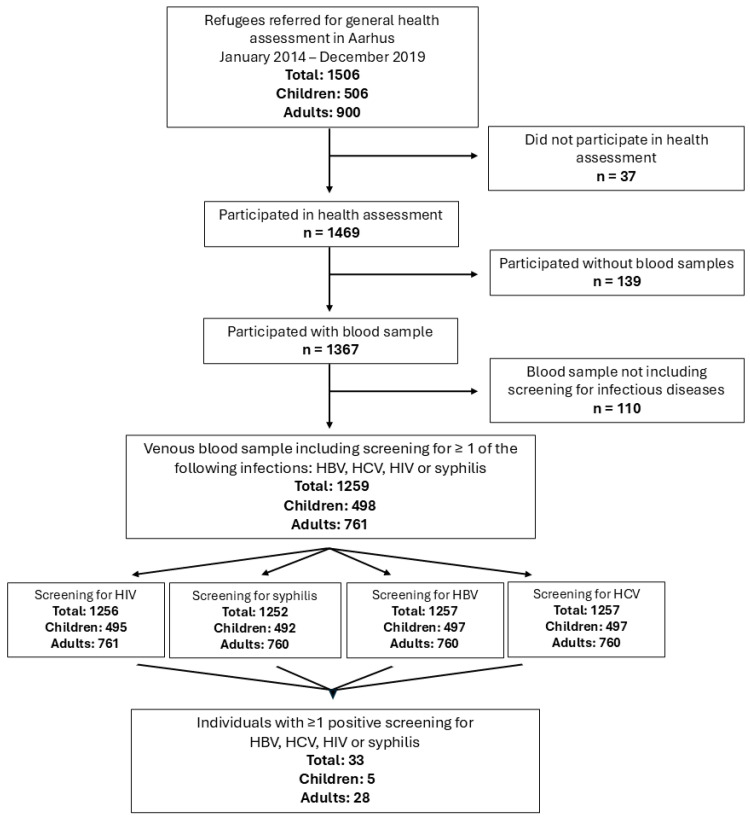
Flowchart depicting the inclusion and screening pathways of refugees following arrival in Denmark during the period January 2014–January 2020.

The total number of individuals screened for infectious diseases, as well as the distribution of positive screening results, is shown in Figure 1. 

Given the small number of positive cases, the results are presented descriptively and no inferential statistical analyses were performed.

Of the 33 participants with a positive screening result, 29 (91%) were referred for specialist evaluation, and 25 (78.1%) attended at least one clinical assessment.

The reasons for non-attendance were inconsistently documented; all non-attenders had screened positive for HCV antibodies but had negative confirmatory HCV-RNA testing, after which no further follow-up was required.

### 3.1. HBV

Fifteen participants (13 adults and two adolescent) screened as positive for HBsAg. All were referred and evaluated at a specialist clinic and diagnosed with chronic inactive HBV infection. Six participants remained in follow-up at the time of inclusion, while eight were discharged from follow-up, most commonly due to repeated non-attendance. Overall, six of the fifteen participants completed follow-up consistent with the intended cascade of care.

One of the identified children with a positive HBV test was identified following a subsequent revision of the study population. However, the authors were unable to retrieve the corresponding medical record for this individual, and therefore the clinical course could not be examined or described in the present study.

### 3.2. HCV

**Figure 2:**
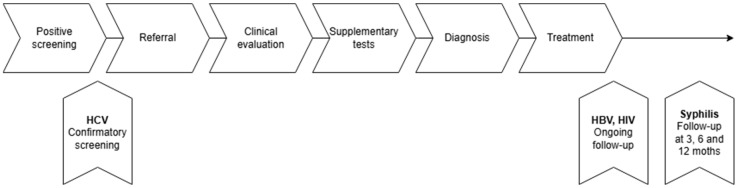
The cascade of care for hepatitis B virus, hepatitis C virus, human immunodeficiency virus and syphilis. HBV (hepatitis B virus), HIV (human immunodeficiency virus), and HCV (hepatitis C virus). The upward arrows indicate specific points in the cascade of care linked to each respective infectious disease.

Ten participants screened positive for HCV antibodies. Confirmatory HCV-RNA testing was positive in three cases and negative in seven. Seven individuals were referred for further evaluation; three attended clinical assessment. One participant completed antiviral treatment with documented adherence and no reinfection, while no participants with confirmed HCV infection completed the full cascade of care as defined in this study (Figure 2).

### 3.3. HIV

One adult participant screened positive for HIV-1 antibodies and was confirmed as HIV positive. The individual was referred, evaluated, and initiated on antiretroviral therapy, with documented attendance at follow-up visits until loss to follow-up later in the study period.

### 3.4. Syphilis

Ten participants (eight adults and two children) screened positive for syphilis and were referred for evaluation. Five individuals initiated treatment, and four completed the treatment as prescribed. Follow-up after treatment was documented in one case only. No participant fulfilled all components of the recommended cascade of care for syphilis (Figure 2).

### 3.5. Interpreters

Among the 27 participants who attended at least one clinical evaluation, an interpreter was present in 20 cases, either in person or via telephone or video. In cases without an interpreter, reasons were variably documented. In one instance, interpreter assistance was limited to part of the consultation.

## 4. Discussion

In this retrospective cohort study focusing on newly arrived refugees screened for selected infectious diseases in a Danish municipal setting, 2.1% of participants had at least one positive screening result for HBV, HCV, HIV, or syphilis. For HBsAg and HCV antibodies, the observed prevalences were 0.9% and 0.7%, respectively (Table 3). When compared with the systematic review by Hahné et al. [16], these estimates correspond more closely to the prevalence reported in the general European population (HBV: 0.1–5.6%; HCV: 0.4–5.2%) than to the higher prevalences previously described among refugee populations (HBV: 1.0–15.4%; HCV: 0–23.4%).

Several factors may contribute to this finding. The so-called “healthy migrant effect” [17], whereby individuals who migrate are often healthier at the time of arrival than both the host population and those remaining in the country of origin, may partially explain the low prevalence observed. In addition, the present cohort differed from many previously studied refugee populations in terms of country of origin, with a predominance of refugees from Syria and neighbouring regions, which is consistent with earlier studies reporting similarly low prevalence estimates among refugees from the Middle East and Western Asia [18,19].

A substantial loss to follow-up was observed among participants with chronic HBV infection, with only six of fourteen individuals completing follow-up consistent with national recommendations. This finding is directly supported by our results and represents one of the central observations of the study. Comparable challenges in continuity of care have been reported previously. A report by Médecins du Monde found that only 26% of undocumented migrants completed treatment or follow-up [20]. However, it should be noted that this population differs substantially from the present cohort of newly arrived refugees with legal residency and formal access to healthcare in Denmark. The comparison therefore serves primarily to illustrate that loss to follow-up is a recurrent challenge across vulnerable populations, rather than to suggest equivalence between settings.

Low adherence to follow-up among individuals with HBV and HCV may partly relate to the largely asymptomatic nature of these infections in early and intermediate stages [21,22]. In the absence of symptoms, patients may underestimate the importance of regular monitoring, despite the risk of long-term complications such as chronic liver disease [23,24]. This underscores the importance of adequate counselling and communication at the time of diagnosis, including clear information about disease progression and follow-up needs, ideally delivered with professional interpreter support. Stigma associated with blood-borne viral infections, often linked to sexual transmission or injection drug use, may further contribute to disengagement from care.

Only one participant screened positive for HIV, corresponding to a prevalence of 0.1%. Although this estimate is based on a single case, it is consistent with findings from other studies involving refugee populations from similar regions of origin [25]. Importantly, this individual was successfully linked to care and initiated on antiretroviral therapy, illustrating that post-arrival screening can facilitate timely HIV diagnosis and treatment even when its prevalence is low.

Syphilis remains a relevant infection to consider as it can remain asymptomatic for prolonged periods, and untreated infection may lead to severe late complications affecting the nervous system and sensory organs [26]. In the present study, wide variation in diagnostic work-up and follow-up was observed, and no participant completed the full recommended cascade of care. These findings suggest a need for clearer standardisation of diagnostic evaluation and follow-up, including the consistent use of WR and RPR as part of syphilis screening protocols among newly arrived refugees in Denmark.

In several cases, the documentation regarding interpreter use was limited. Among participants attending clinical evaluation, 26% did not have a documented interpreter present at the first consultation. While documentation quality does not directly determine patient outcomes, the absence of professional interpreter support represents a potential risk to effective communication and patient safety, as misinterpretation may lead to the misunderstanding of diagnoses, inadequate treatment, or poor adherence [27,28,29]. Improved documentation may help identify structural gaps and support quality improvement initiatives.

### Strengths and Limitations

The strengths of this study include its six-year observation period and the inclusion of all newly arrived refugees undergoing systematic post-arrival screening in a large Danish municipality. In addition, the use of Denmark’s national electronic health record system allowed for the linkage of screening results with subsequent clinical management at the individual level.

Several limitations should be acknowledged. First, data were derived exclusively from electronic medical records, limiting insight into patient-reported barriers and reasons for disengagement from care. Second, heterogeneity in clinical practice—particularly in the management of syphilis across medical specialties—complicated the assessment of the adherence to guidelines. Moreover, incomplete documentation regarding interpreter use may have led to misclassification in some cases. An additional limitation relates to the very low number of participants with positive screening results. While this reflects a genuine finding of low disease prevalence, it limited the level of detail with which results could be presented and precluded meaningful subgroup analyses. Moreover, given the small number of positive cases within a well-defined local cohort, particular care was required to protect participant anonymity and comply with data protection regulations. As a consequence, data presentation was necessarily aggregated, and some potentially informative clinical characteristics could not be reported.

The descriptive nature of the analysis should be interpreted in light of these constraints. The study was not designed to assess associations or predictors of loss to follow-up, but rather to document progression through the cascade of care and identify critical points of attrition following post-arrival screening.

A subsequent revision of the study population identified one additional child with a positive HBV test who was included in the statistical analyses; however, the corresponding medical record could not be retrieved, and the individual clinical course could therefore not be examined or described.

## 5. Conclusions

In this local Danish cohort of newly arrived refugees, the prevalence of HBV, HCV, HIV, and syphilis identified through post-arrival screening was low and consistent with findings from comparable settings. While screening effectively enabled case identification, the principal finding of this study was substantial attrition at multiple stages of the cascade of care following a positive screening result.

In particular, loss to follow-up among individuals with chronic infections—most notably HBV—significantly limited the clinical impact of screening. These findings indicate that the effectiveness of post-arrival screening programmes depends not only on diagnostic testing but also on the healthcare system’s capacity to ensure sustained engagement in follow-up and treatment.

Targeted efforts to strengthen continuity of care, including clearer follow-up pathways, improved coordination across services, and the consistent use of professional interpreters, are needed to address these gaps. Future research should focus on identifying structural and individual barriers to retention in care and on developing interventions that support equitable and effective follow-up for refugees diagnosed through post-arrival screening.

## Figures and Tables

**Table 1:** National guidelines for the further evaluation of patients with a positive screening for hepatitis B virus (HBV), hepatitis C virus (HCV), human immunodeficiency virus (HIV), and syphilis.

Positive Screening	Procedure According to Guidelines
HBV	Supplementary tests Test for HBV-DNA two times. Liver specific objective clinical examination. Ultrasound and two fibro scans (on the same level) of the liver. Biopsy (if significant variation is seen in the two fibro scans). Liver-specific blood samples (ALAT, bilirubin, alkaline phosphatase, albumin, α-fetoprotein, and coagulation factor II, VII and X). Screening for co-infections HIV. HCV and HDV
HCV	Supplementary tests HCV-RNA detection by PCR and determination of genotype. Liver-specific clinical examination. Ultrasound and two fibro scans (on the same level) of the liver. Liver-specific blood samples (ALAT, bilirubin, alkaline phosphatase, albumin, α-fetoprotein, and coagulation factor II, VII and X). Screening for co-infections HIV. HBV.
HIV	Supplementary tests Confirmatory test (positive HIV Ag/Ab test). CD4 cell count. HIV-RNA detection and viral load. Anamnestic information on time of infection and transmission route. Resistance level. Screening for co-infections HAV, HBV and HCV. Syphilis. Tuberculosis. Toxoplasmosis. CMV.
Syphilis	Supplementary tests Extended serology: quantitative levels of anti-lipoidale antibodies (WR + RPR) and anti-treponemal antibodies (T. Pallidum IgM and IgG). Clinical examination with focus on skin (roseola and syphilitic gummata), genitals, CNS and the oral cavity (syphilis chancroids). PCR-tests (wound swabs) and dark field microscopy Screening for co-infections HIV.

HBV (hepatitis B virus), HIV (human immunodeficiency virus), HCV (hepatitis C virus), HDV (hepatitis D virus), PCR (polymerase chain reaction), Ag/Ab test (antigen/antibody test), HAV (hepatitis A virus), CMV (cytomegalovirus), WR + RPR (Wassermann reaction + rapid plasma reagin), and CNS (central nervous system).

**Table 2:** Demographics of the study population arriving in Aarhus, Denmark from January 2014 to January 2020.

Region/Country of Origin	Participants (Children < 18 Years) *n*, (*n*)	Median Age Years [Range]	Female *n* (%)	Type of Migration Asylum/Family Reunited/UNHCR ^1^/Unknown *n* (%)
**Western Asia**	1011 (408)	24 [0–76]	472 (46.7)	615 (60.8)/382 (37.8)/ 14 (1.4)/0
Syria	967 (402)	23 [0–76]	447 (46.2)	593 (61.3)/365 (37.7)/ 9 (0.9)/0
Lebanon	18 (4)	32 [6–60]	11 (61.1)	9 (50)/7 (38.9)/ 2 (11.1)/0
Iraq	17 (2)	24 [5–32]	10 (58.8)	11 (64.7)/5 (29.4)/ 1 (5.8)/0
Other	9 (0)	46 [26–71]	4 (44.4)	2 (22.2)/5 (55.5)/ 2 (22.2)/0
**Eastern Africa**	202 (77)	21 [0–69]	86 (42.6)	115 (56.9)/86 (42.6)/ 1 (0.5)/0
Eritrea	123 (38)	22 [0–65]	47 (38.2)	81 (65.7)/42 (34.1)/ 0/0
Somalia	59 (32)	16 [1–69]	28 (47.5)	21 (35.6)/37 (62.7)/ 1 (1.7)/0
Ethiopia	19 (7)	25 [6–36]	11 (57.9)	12 (63.2)/7 (36.8)/ 0/0
Other	1 (0)	19	0	1 (100)/0/ 0/0
**Southern Asia**	193 (78)	22 [0–75]	78 (40.4)	161 (83.4)/31 (16)/ 0/1 (0.5)
Iran	121 (39)	27 [0–63]	53 (43.8)	105 (86.8)/16 (13.2)/ 0/0
Afghanistan	71 (39)	17 [0–75]	25 (35.2)	55 (77.5)/15 (21.1)/ 0/1 (1.4)
Other	1 (0)	16	0	1 (100)/0/ 0/0
**Middle Africa**	52 (27)	16 [0–75]	31 (59.6)	0/0/ 52 (100)/0
Congo	37 (18)	19 [0–75]	22 (59.5)	0/0/ 37 (100)/0
Central Africa Republic	15 (9)	14 [2–74]	9 (60.0)	0/0/ 15 (100)/0
**South America**	9 (3)	18 [4–49]	7 (77.8)	0/0/ 9 (100)/0
Columbia	9 (3)	18 [4–49]	7 (77.8)	0/0/ 9 (100)/0
**Other ^2^**	9 (1)	34 [1–40]	4 (44.4)	7 (77.8)/2 (22.2)/ 0/0
**Unknown**	30 (11)	25 [1–69]	18 (60.0)	8 (26.7)/11 (36.7)/ 3 (10.0)/8 (26.7)
**Total**	**1506 (606)**	**22 [0–76]**	**696 (46.2)**	**906 (60.2)/512 (33.4)/** **79 (5.2)/9 (0.6)**

^1^ Quota refugees arriving through the UN Refugee Agency; ^2^ countries represented by less than five participants: Myanmar, Morocco, Libya, Egypt, Russia, Kuwait, Palestine, Jordan, Zambia, and Pakistan

**Table 3:** Rates of positive screenings in 1506 refugees arriving in Aarhus, Denmark from January 2014 to January 2020.

	Hepatitis B Virus	Hepatitis C Virus	Human Immunodeficiency Virus	Syphilis	Total
Positive screening *n* (% of entire study population)	15 (1.0)	10 (0.7)	1 (0.1)	10 (0.7)	33 (2.2)
Arrived at follow-up *n* (% of all positive screenings)	6 (18)	No follow-up after treatment	1 (0.3)	1 (0.3)	7 (21)
Initiation of treatment	No treatment ^1^	1 (0.3)	1 (0.3)	5 (15)	7 (21)

^1^ All patients who screened positive for hepatitis B virus were not treated, as they were diagnosed with chronic inactive infection and therefore had no indication for treatment.

## Data Availability

Data was stored in secure databases RedCap. (Vanderbilt, United States of America) system, such as demographic data, interview results and blood sample results.

## Abbreviations

The following abbreviations are used in this manuscript:

EPJ	Electronic patient journal
HAV	Hepatitis A virus
HBV	Hepatitis B virus
HCV	Hepatitis C virus
HDV	Hepatitis D virus
HIV	Human immunodeficiency virus
PCR	Polymerase chain reaction
DNA	Deoxyribonucleic acid
RNA	Ribonucleic acid
